# Perinatal mortality and its association with antenatal care visit, maternal tetanus toxoid immunization and partograph utilization in Ethiopia: a meta-analysis

**DOI:** 10.1038/s41598-021-98996-5

**Published:** 2021-10-04

**Authors:** Melaku Desta, Tadesse Yirga Akalu, Yoseph Merkeb Alamneh, Asmare Talie, Addisu Alehegn Alemu, Zenaw Tessema, Desalegn Yibeltal, Alehegn Aderaw Alamneh, Daniel Bekele Ketema, Wondimeneh Shibabaw Shiferaw, Temesgen Getaneh

**Affiliations:** 1 Department of Midwifery, College of Health Sciences, Debre Markos University, Debre Markos, Ethiopia; 2 Department of Nursing, College of Health Sciences, Debre Markos University, Debre Markos, Ethiopia; 3 Department of Biomedical Sciences, College of Medicine, Debre Markos University, Debre Markos, Ethiopia; 4 Department of Pharmacy, College of Health Sciences, Debre Markos University, Debre Markos, Ethiopia; 5 Department of Human Nutrition and Food Science, College of Health Sciences, Debre Markos University, Debre Markos, Ethiopia; 6 Department of Public Health, College of Health Sciences, Debre Markos University, Debre Markos, Ethiopia; 7 Department of Nursing, College of Health Science, Debre Berhan University, Debre Markos, Ethiopia

**Keywords:** Diseases, Health care, Risk factors

## Abstract

Despite remarkable progress in the reduction of under-five mortality; perinatal mortality is the major public health problem in Africa. In Ethiopia, the study findings on perinatal mortality and its predictors were inconsistent. Therefore, this systematic review and meta-analysis estimated the pooled perinatal mortality, and its association with antenatal care visit, maternal tetanus toxoid immunization, and partograph monitoring. International databases like PubMed, SCOPUS, Google Scholar and Science Direct were systematically searched. I squared statistics was used to determine the levels of heterogeneity across studies and the pooled estimate was computed using a random-effect model. The meta-analysis showed that a pooled prevalence of perinatal mortality in Ethiopia was 6.00% (95% CI 5.00%, 7.00%). The highest proportion of perinatal mortality was a stillbirth, 5.00% (95% CI 4.00%, 7.00%). Women who had antenatal care visit [OR = 0.20 (95% CI 0.12, 0.34)], maternal tetanus toxoid immunization [OR = 0.43 (95% CI 0.24, 0.77)] and partograph monitoring [POR = 0.22 (95% CI 0.06, 0.76)] reduced the risk of perinatal mortality. Whereas, previous history of perinatal mortality [POR = 7.95 (95% CI 5.59, 11.30)] and abortion history (POR = 2.02 (95% CI 1.18, 3.46)) significantly increased the risk of perinatal mortality. Therefore, antenatal care visit, maternal tetanus toxoid vaccination uptake, and partograph utilization should be an area of improvements to reduce perinatal mortality.

## Background

Perinatal mortality consists of stillbirth (pregnancy losses occurring at or after 28 weeks of gestation) and early neonatal deaths (deaths to live births within the first seven days of life)^1^. According to the recent Global Burden of Disease estimation 4.5 million deaths (2.1 million stillbirths and 2.4 million early neonatal deaths) were occurred in 2015 worldwide^2^. One million neonatal mortality occurred on the first day and close to one million dying within the first six days^3^. The burden of perinatal mortality was disproportional across the world. Low and middle-income countries (LMICs), mainly Sub Saharan Africa (SSA) take the lion’s share of the burden^4^. In developing countries, the risk of death in the perinatal period is much greater than in developed countries^5^. A systematic review done in SSA showed that the pooled estimate of perinatal mortality was 34.7 per 1000 live births^6^. The rate of perinatal mortality of Ethiopia is among the highest in SSA, which was 90 per 1000 in a hospital setting and 40 per 1000 live births in the community setting^7^ and 33 deaths per 1000 live births based on the reports of demographic Health Survey of 2016^8^.

Despite a significant change was made in child health^5^, the reduction in perinatal mortality reduction is slower which remains a major public health concern in developing countries^2^. Thus, on current trends, more than 60 countries (including Ethiopia) will miss the Sustainable Developmental Goals (SDGs) target of reducing neonatal mortality by 12 deaths per 1000 live births in 2030^9^. Perinatal mortality is a key indicator for the quality of prenatal, intrapartum and newborn care, considered as basic indicators of a country’s socio-economic situation and quality of life^10^. Moreover, it is a proxy measure of maternal health status and has enormous economic, social and health implications for families and society^11^. Evidence also confirmed that perinatal death greatly increased the vulnerability of women to experience negative psychological and social consequences^12^.

The maternal and perinatal health service such as antenatal care service, maternal immunization status, skilled birth delivery and sociodemographic factors as educational status of women and husband, wealth, residence, and obstetrical factors as obstetric related disorder during pregnancy, history of previous adverse perinatal outcomes, birth preparedness; and neonatal related like preterm birth, low birth weight and neonatal sepsis are associated with perinatal mortality^13–16^. While preterm birth, infection, hypertensive disease and intrapartum asphyxia accounted for the most common causes of perinatal mortality in LMICs^17^. Besides, systematic review done showed that low socioeconomic status, lack of quality health-care services, obstetric complications and lack of antenatal care were the most factors of perinatal mortality^18^. However, evidence suggests that two-third of perinatal mortality could be prevented using interventions including perinatal care^19^.

Despite, perinatal mortality can be prevented using simple cost-effective antenatal and intrapartum interventions as antenatal care visit and partograph utilization; the rate of perinatal mortality remains a major challenge in Ethiopia and associated with maternal depression^20,21^. Thus, to end this, the modifiable factors have to be improved permanently and universally to reduce perinatal mortality. But inconsistent and inconclusive evidence was found on the prevalence of perinatal deaths and its association with antenatal care visit, maternal tetanus vaccination, partograph utilization and poor obstetric history; which cannot be used for policymakers at the national level. Therefore, this systematic review and meta-analysis aimed to estimate the pooled national prevalence of perinatal mortality and its association with antenatal care visit, maternal tetanus vaccination status, partograph monitoring of labour and poor obstetric history. This evidence is essential to inform the health policymakers, program implementers and maternal, neonatal and perinatal health experts to achieve the SDGs target of ending preventable perinatal deaths in 2030.

## Methods

### Study design, data sources and search strategies

A systemic review and meta-analysis of published studies were conducted to estimate the prevalence of perinatal mortality and its association with antenatal care visit, maternal tetanus toxoid immunization and partograph utilization in Ethiopia. This systematic review and meta-analysis was reported according to the Preferred Reporting Items for Systematic Review and Meta-Analysis (PRISMA) 2009 statement checklist^22^ (Supplementary file 1). All relevant published studies were searched from the major international databases like PubMed, Cochrane Library, Web of science, science direct, and African Journals Online databases. Google Scholar and Google hand searches were also used. Additionally, a search was made for the reference list of studies already identified to retrieve additional articles. Studies identified through the systematic search were retrieved and managed using Endnote X7. The Population, Exposure, Comparison and Outcomes (PECO) search formula was used to retrieve articles.

*Population* All births from the 28 weeks of gestation till the 7 days’ postpartum period in Ethiopia were the population of interest.

*Exposures* Predictors of perinatal mortality included antenatal care visit, maternal toxoid injection, partograph monitoring of labour, previous perinatal death and abortion.

Comparisons were defined for each predictor based on the reported reference group for each predictor in each respective variable.

*Outcome* Perinatal mortality.

For each of the selected components of PECO, electronic databases were searched using the keyword search and the medical subject heading [MeSH] words. Key words like perinatal mortality, stillbirth, predictors, determinants, antenatal care visit, maternal tetanus toxoid injection, partograph monitoring as well as Ethiopia were used. The search terms were combined by the Boolean operators "OR" and "AND.

### Study selection and eligibility criteria

All published studies published from 2010 until the end of our search in the English language (7/4/2020) were retrieved to assess eligibility for inclusion in this review and critical assessment. The article selection underwent several steps. Two reviewers (MD and TYA) evaluated the retrieved articles for inclusion using their title, abstract and full-text review. Any disagreement during the selection process between the reviewers was resolved by consensus. Full texts of selected articles were then evaluated using the *prior* eligibility during the encounter of duplication; only the full-text article was retained. The included studies that were reported perinatal mortality or AND predictors, antenatal care visit, maternal tetanus toxoid injection, partograph monitoring and previous perinatal death. All prospective and retrospective cohort studies, cross-sectional studies, case–control and Demographic and Health Survey (DHS) reports were included in this review. However, this review excluded studies that were case reports of populations, abstracts of conferences, articles without full access and the outcome of interest not reported. Studies that were not fully accessed after at least two email contacts of the principal investigator were excluded.

### Outcome variable

The primary outcome of this systematic review and meta-analysis was perinatal mortality in Ethiopia. The secondary outcomes were: the pooled effect of selected predictors on perinatal mortality, antenatal care visit, maternal toxoid injection, partograph monitoring of labour, previous history of perinatal death and abortion.

Poor obstetric history means previous unfavorable fetal outcome in terms of those having at least one of the following two or more consecutive spontaneous abortions, history of intrauterine fetal death, intrauterine growth retardation, stillbirth, early neonatal death, and/or congenital anomalies.

Maternal stress is the exposure of an expectant mother to stress, which can be caused by stressful life events like or by environmental hardships, which resulting changes to the mother's hormonal and immune system may harm the fetus's (and after birth, the infant's) immune function and brain development. Thus, women who endorse life events experienced in the prior 6 months across 11 domains, including financial, legal, relationships, career, safety in the community, safety in the home, medical issues about others, medical issues about self, authority, home issues, and prejudice. Pregnant women who endorsed the event as positive, negative, or neutral. The numbers of domains with at least one negative event were summed to create a negative life events domain score, with higher scores suggesting increased maternal stress^23^.

## Data extraction and quality assessment

Two reviewers (MD and TYA) independently performed data extraction using a predefined eligibility criterion and assessed articles for overall study quality. The standardized data extraction format was prepared using a Microsoft Excel spreadsheet. The data extraction format included primary author, year of publication, and region of the study, sample size, and the reported outcome (perinatal mortality) and the number of cases or live birth who developing the respective outcome, and the selected predictors of perinatal mortality mainly its association with antenatal care visit, maternal tetanus toxoid vaccination, partograph monitoring of labour and previous perinatal history; previous abortion and previous perinatal mortality were extracted.

The quality of included studies was assessed using the Newcastle–Ottawa Scale (NOS) quality assessment tool based on the three components; the selection of the study groups, comparability of the study groups, and ascertainment of exposure or outcome^24^. The main component of the tool was graded from five stars and mainly emphasized the methodological quality of each primary study. The other component of the tool was graded from two stars and mainly concerned with the comparability of each study and the last component of the tool graded from three stars and was used to evaluate the results and statistical analysis of each original study. The NOS included three categorical criteria with a maximum score of 9 points. The quality of each study was assessed using the following score algorithms: ≥ 7 points were considered as “good”, 4 to 6 points were considered as “moderate” and ≤ 3 points were considered as “poor” quality studies. Consequently, to improve the validity of this systematic review result, only primary studies of fair to good quality have been included.

### Heterogeneity and Publication bias

The publication bias was assessed using Egger’s^25^ and Begg’s^26^ tests with a *p* value of less than 0.05. I squared statistic was used to assess heterogeneity across studies and a *p* value of less than 0.05 was used to detect heterogeneity. As a result of the presence of heterogeneity, a random-effects model was used as a method of analysis^27^ resulting in the use of a random-effects meta-analysis model to estimate the pooled effect based on the metaprop software by STATA version 14 of the double arcsine transformations^28^. Because, the proportions contain inadmissible values especially when the statistic is near the boundary and computation of confidence intervals is not possible when the statistic is on the boundary, as the estimated standard error is set to zero and as a consequence, the metan command automatically excludes studies with proportion equal to 0 or 1 from the pooled estimate.

### Statistical methods and analysis

The extracted data in Microsoft Excel were exported to Stata version 14 for analysis. Metaprop Stata command with double arcsine transformations was used to estimate the pooled prevalence^28^. Because, the proportions contain inadmissible values especially when the statistic is near the boundary and computation of confidence intervals is not possible when the statistic is on the boundary, as the estimated standard error is set to zero and as a consequence, the metan command automatically excludes studies with proportion equal to 0 or 1.

Forest plots and Odds Ratios were used to present the pooled estimate with 95% confidence intervals (CI). Subgroup analysis was conducted based on the geographic region, and study design. Besides, a meta-regression model based on sample size, geographic region, study setting, design and year of publication was used to identify the sources of random variations in the included studies. The effect of selected determinant variables was analyzed using separate categories of meta-analysis^29^. Also, we conducted a sensitivity analysis to assess whether the pooled prevalence estimates were influenced by individual studies.

## Results

### Study identification and characteristics of included studies

**Figure 1:**
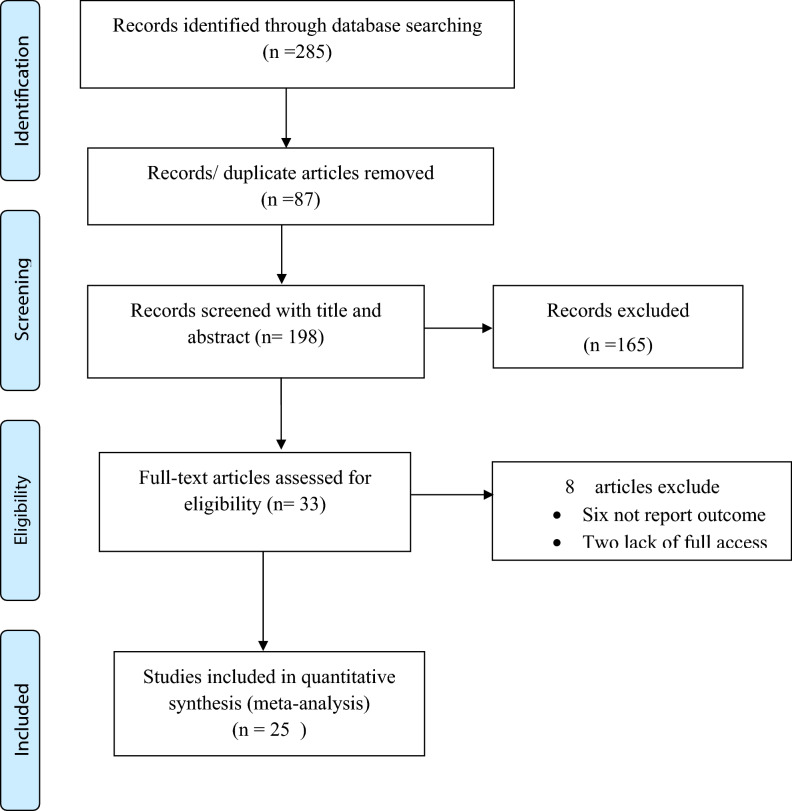
PRISMA flow diagram of the included studies in the systematic review and meta-analysis to estimate perinatal mortality in Ethiopia.

This systematic review and meta-analysis included published studies on the pooled prevalence of perinatal mortality in Ethiopia using international electronic databases. A total of 285 data sources were found in the review. Of these, 87 duplicated records were deleted and 165 articles were excluded by the screening of titles and abstracts. Subsequently, a total of 33 full-text papers were assessed for eligibility based on the inclusion and exclusion criteria. Thus, six studies were excluded due to not report the outcome of interest^30–35^ and the remained two articles excluded due to lack of access to the full text^36,37^. Finally, 25 studies have been included in the final quantitative meta-analysis (Fig. 1).

Of the 25 studies included in the final quantitative meta-analysis, 16, 13 and 21 studies were used to estimate the overall pooled prevalence of perinatal mortality, early neonatal death and stillbirth with total live birth of 91,370, 49,163 and 71,579 for each outcome respectively. In regards to the design of the included studies, two were surveys^38^, six prospective studies^39–44^, and twelve cross-sectional and four case–control studies. Of those, 16 (64%) of included studies were community-based studies. The articles were published between 2012 and 2020. The largest sample size was 48,994 of live births in a national level study^45^ and the smallest sample, 300 live births, from one geographic region were conducted at Southern Nations, Nationalities and Peoples Representative (SNNPR). All studies were conducted in the five geographic regions of Ethiopia. Six studies (24%) were from Amhara, seven (28%) were from SNNPR, four (16%) were from Oromia, three (12%) were from Tigray, two were the national-level studies and the remaining two was at Harar and Addis Ababa (Table 1). The included studies have fair to good qualities based on the NOS assessment tool (Supplement-2).

**Table 1:** Characteristics of included studies in the systematic review and meta-analysis, Ethiopia.

Authors	Year	Region	Setting	Design	Sample	Outcome	Main findings
Tessema et al.^45^	2016	National	Community based	Survey	48,994	PM	1336 PM
W/Amanuel and Gelebo^46^	2019	Tigray	Facility based	Cross-sectional	2738	PM, END and stillbirth	170 PM, 111 END and 60 stillbirths
Aragaw^47^	2016	Oromia	Facility based	Cross-sectional	3782	PM, END and stillbirth	372 PM perinatal mortality, 104 END and 268 stillbirths
Roro et al.^48^	2018	Oromia	Community based	Nested case control	4438	PM, END and stillbirth	73PM, 47 END and 26 stillbirths
Tesfaye	2019	AA	Facility based	case control	3160	PM, END and stillbirth	241 PM, 60 END and 181 stillbirths
Mihretu et al	2017	SNNPR	Facility based	Cross-sectional	300	Perinatal mortality	52 PM
Andargie et al.^39^	2013	Amhara	Community based	Prospective cohort	1752	PM, END and stillbirth	88 PM, 47 END and 41 stillbirths
Yirgu et al.^49^	2016	Amhara	Community based	Nested case control	4097	PM, END and stillbirth	102PM, 45 END and 57 stillbirths
Tura et al.^40^	2020	Harar	Facility based	Prospective cohort	7929	PM, END and stillbirth	615 PM, 17 END and 598 stillbirths
Debelew et al.^41^	2014	Oromia	Community based	Prospective cohort	3510	PM, END and stillbirth	123 PM, 76 END and 47 stillbirths
Limaso et al.^42^	2020	SNNPR	Community based	Prospective cohort	586	PM, END and stillbirth	29 PM, 15 END and 14 stillbirths
Eyowas et al	2016	Amhara	Facility based	Cross-sectional	3003	PM, END and stillbirth	318 PM, 13 END and 305 stillbirths
Lolaso et al	2019	SNNPR	Facility based	Cross-sectional	718	PM, END and stillbirth	25 PM, 1 END and 24 stillbirths
Mehari et al	2020	Tigray	Facility	cross-sectional	752	PM, END and stillbirth	50PM, 6 END and stillbirth
Bayou and Berhan^50^	2012	SNNPR	Facility based	Case control	5030	PM, END and stillbirth	432 PM, 58 END and 394 stillbirths
Tsegaye and Kassa^51^	2018	SNNPR	Facility based	cross-sectional	580	PM	16 PM
Desta et al.^43^	2016	Oromia	Facility based	Prospective cohort	7367	Early neonatal death	144 early neonatal death
Lakew et al.^52^	2017	Amhara	Facility based	Cross-sectional	2555	Stillbirth	218 stillbirths
Adane et al.^53^	2014	Amhara	Facility based	Cross-sectional	481	Stillbirth	24 stillbirths
Berhe et al.^38^	2017	National	Community based	Survey	12,560	Stillbirth	Survey
Assefa et al.^44^	2012	SNNPR	Community based	Prospective cohort	1438	Stillbirth	Community prospect
Adhena et al.^54^	2017	Tigray	Facility based	Cross-sectional	425	Stillbirth	41 stillbirths
Abdo et al	2017	SNNPR	Facility based	Cross-sectional	327	Stillbirth	28 stillbirths
Cherie and Mebratu	2017	Amhara	Facility based	Cross-sectional	462	Stillbirth	38 stillbirths
Zerfu et al.^55^	2016	Oromia	Community based	cohort study	432	Stillbirth	19 stillbirths

PM: Perinatal mortality, END: early neonatal death.

### Meta-analysis of perinatal mortality in Ethiopia

**Figure 2:**
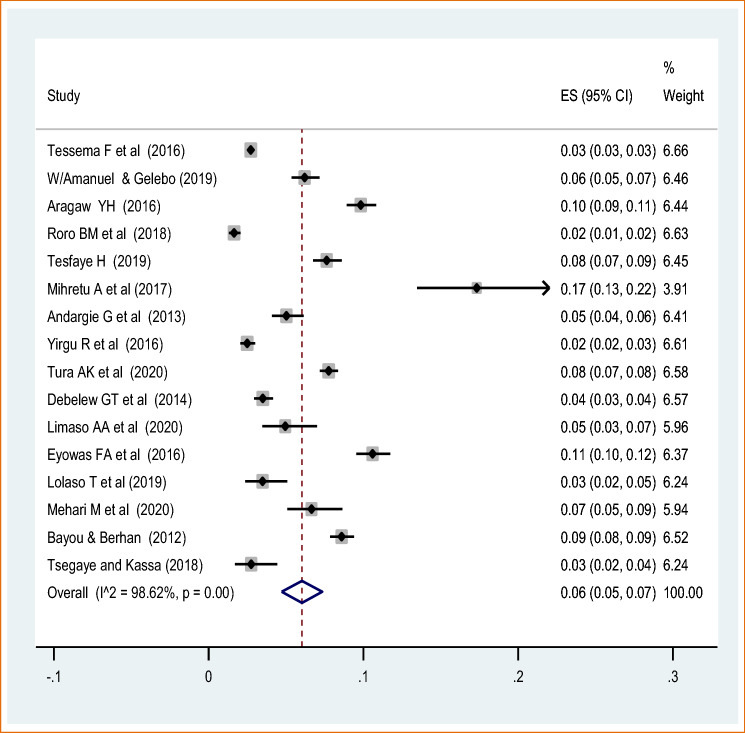
Forest plot on the pooled prevalence of perinatal mortality in Ethiopia: meta-analysis.

The highest perinatal mortality was reported at the national level survey^45^ while the lowest was 6 per 580 live births reported from SNNPR^51^. The meta-analysis of 16 studies showed that the pooled national prevalence of perinatal mortality was 6% (95% CI 5.00, 7.00). A random-effect model was used due to significant heterogeneity (I^2^ = 97.9%, *p* value < 0.05) (Fig. 2). There was a publication bias based on the Eggers test with a *p* value of 0.0002. Due to the presence of significant publication bias, we conducted trimmed and filled analysis. The univariate meta-regression model was also used to identify possible sources of heterogeneity. However, none of these variables was found to be statistically significant, *p* value > 0.05. Moreover, the sensitivity analysis using a random-effects model showed that no single study had unduly influenced the overall estimate of the perinatal mortality (Supplementary file 2).

**Figure 3:**
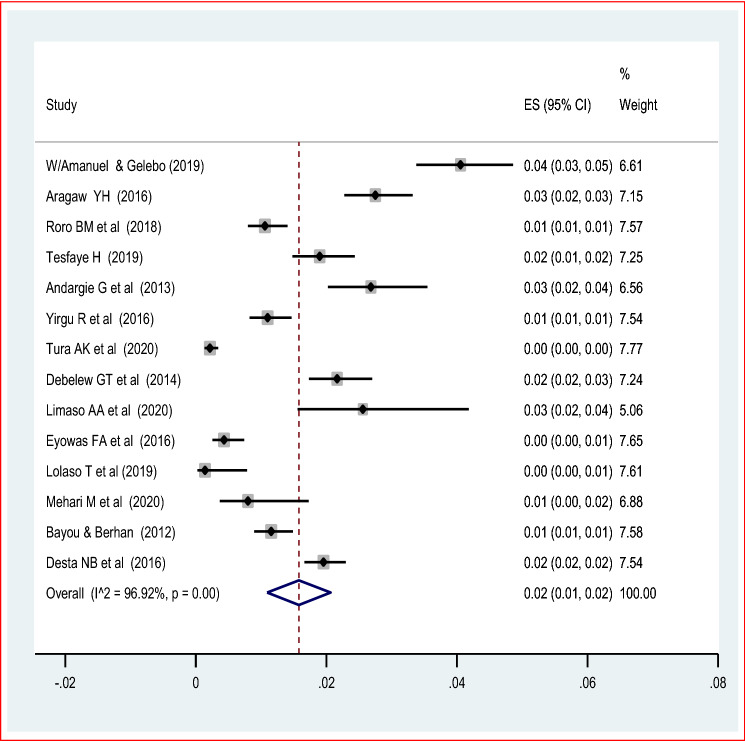
Forest plot on the pooled prevalence of early neonatal death in Ethiopia: meta-analysis.

**Figure 4:**
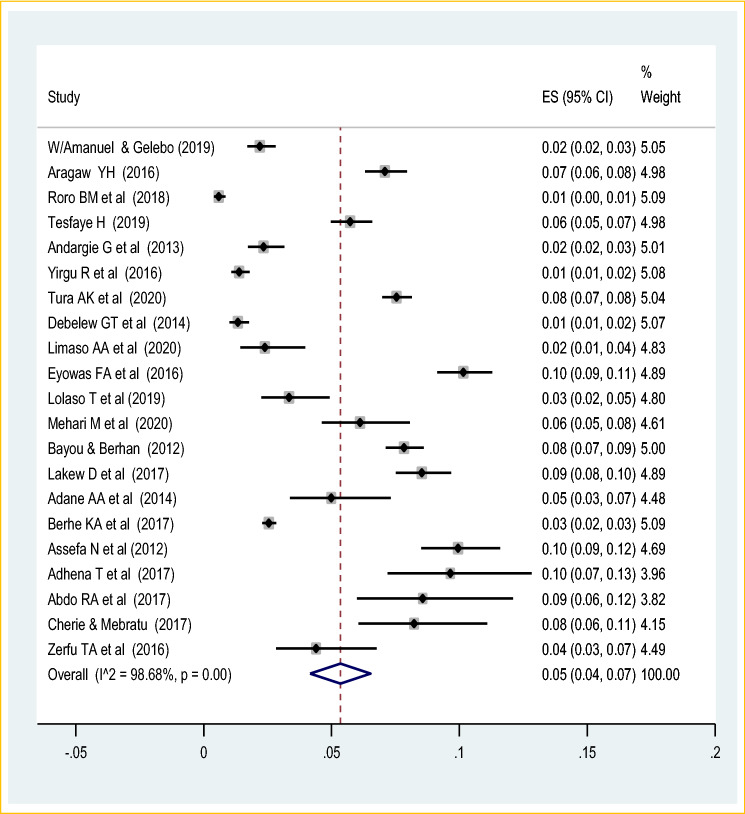
Forest plot on the pooled prevalence of stillbirth in Ethiopia: meta-analysis.

The highest cases of early neonatal mortality and stillbirth were 144 per 7367 occurred at Oromia region^43^ and 588 cases per 7929 live births of cohort study at Harar^40^. The pooled random-effect meta-analysis of fourteen studies found that the pooled prevalence of early neonatal death among live births was 2% (95% CI 0.01, 0.02). The random-effect model was used due to the presence of significant heterogeneity (I^2^ = 96.9%, *p* value < 0.05) (Fig. 3). There is no publication bias with the Eggers and Beggs test (*p* value of 0.35 and 0.65 respectively). The meta-analysis of twenty-one studies also showed that the pooled prevalence of stillbirth was 5% (95% CI 3.00, 6.00). The random-effect model was used due to the presence of heterogeneity (I^2^ = 98.7%, *p* value < 0.05) (Fig. 4). Therefore, to identify the source of heterogeneity a univariate regression was done based on the year of publication, sample size, geographic region, the study design and setting. But, none of the variables was significant. The funnel plot observation also showed that there is a symmetrical distribution and the objective assessment of the egger’s and Begg’s test revealed that there was publication bias with a *p* value of 0.001 and 0.034. Thus, the Duval and trimmed full analysis were performed to control bias.

## Subgroup analysis

The subgroup analysis was conducted based on the region, the study setting and design for perinatal mortality and stillbirth. Therefore, this random effect meta-analysis based on the geographic region revealed that the highest pooled prevalence of perinatal mortality was observed in the Harar, 8.00 (95% CI 7.00, 9.00) followed by SNNPR, 7.00% (95% CI 7.00% (95% CI 4.00, 10.0) and lowest occurred at the national level study, 3.00% (95% CI 2.00, 4.00) respectively (Table 2). Also, the pooled subgroup analysis showed that the perinatal mortality was highest in the facility-based studies, 8.00% (95% CI 6.00, 9.00) and in the cross-sectional studies, 8.00% (95% CI 5.00, 10.0). The subgroup analysis of this meta-analysis showed that the highest pooled prevalence of stillbirth among live births was, 8.00% (95% CI 12.8, 28.63) and the highest prevalence of stillbirth, 7.00% (95% CI 5.00, 9.00) was reported in facility-based studies based on the thirteen studies based on the random-effect model (Table 2).

**Table 2:** Subgroup analysis of perinatal mortality and stillbirth by geographic region in Ethiopia.

Outcome	Category	No of studies	Prevalence [95%CI]	I^2^	*p* value
Perinatal mortality by region	Tigray	2	6.00 (5.00,7.0)	0%	< 0.0001
	Oromia	3	5.00 (1.00,9.00)	0%	< 0.0001
	SNNPR	5	7.00 (4.00,10.0)	96.2%	< 0.0001
	Amhara	3	6.00 (1.00,11.0)	0%	< 0.001
	Addiss Abeba	1	6.00 (5.00,7.00)	–	–
	National	1	3.00 (2.00,4.00)	–	–
	Harar	1	8.00 (7.00,9.00)	–	–
Perinatal mortality by study setting and design	Community based	6	3.00 (2.00,4.00)	92.2	< 0.0001
	Facility based	10	8.00 (6.00,9.00)	94.7%	< 0.0001
	Cross-sectional	7	8.00 (5.00,10.0)	96.4%	< 0.0001
	Cohort	4	6.00 (3.00,8.00)	85.6%	< 0.0001
	Case control	4	5.00 (2.00,8.00)	99.1%	< 0.0001
	Survey	1	3.00 (2.00,4.00)	–	–
Stillbirth	Tigray	3	6.00 (3.00,10.0)	0%	< 0.0001
	Oromia	4	3.00 (1.00,5.00)	98.7%	< 0.0001
	SNNPR	5	6.00 (3.00,9.00)	95.9%	< 0.0001
	Amhara	6	6.00 (3.00,9.00)	95.7%	< 0.001
	Addiss Abeba	1	6.00 (5.00,7.00)	–	–
	National	1	3.00 (2.00,3.05)	–	–
	Harar	1	8.00 (7.00,9.00)	–	–
Stillbirth by study setting	Facility based	13	7.00 (5.00,8.00)	97.2%	< 0.001
	Community based	8	3.00 (2.00,4.00)	96.5%	< 0.001

## Impact of antenatal and intrapartum interventions to reduce perinatal mortality

The impact of antenatal interventions (antenatal care visit, maternal immunization) and intrapartum (partograph monitoring of labour) interventions the simplest interventions were assessed and revealed that those interventions are modifiable predictors that reduced the risk of perinatal mortality based on the pooled effect meta-analysis.

**Figure 5:**
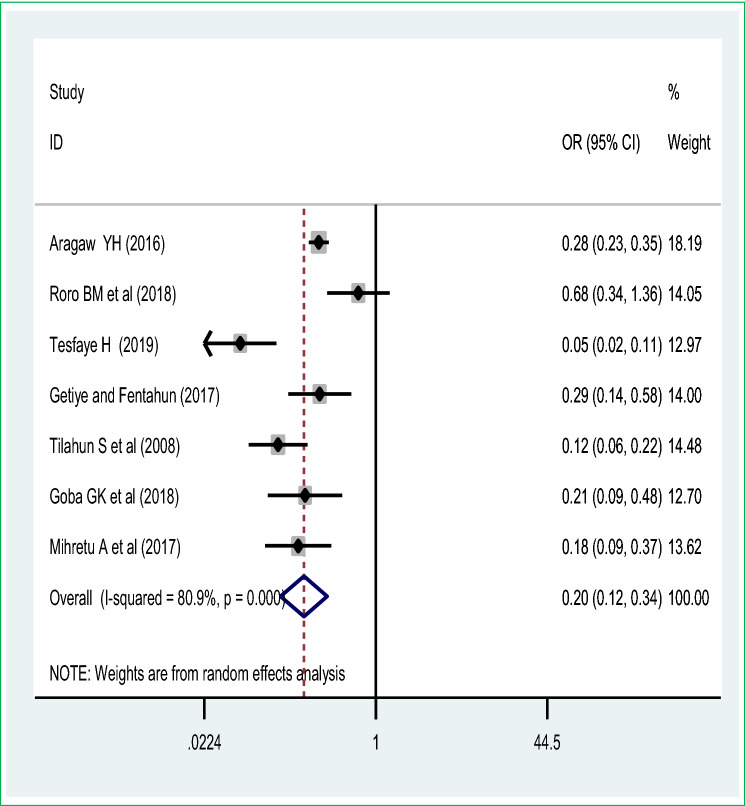
Pooled odds ratio on the association of antenatal care visit and perinatal mortality.

**Figure 6:**
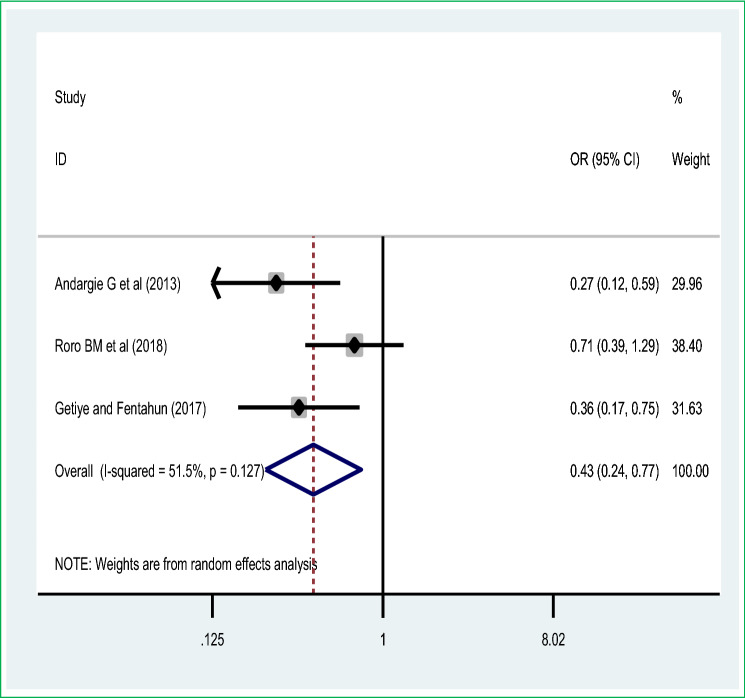
Association of maternal tetanus toxoid immunization on perinatal mortality meta-analysis.

The pooled effect of seven studies showed that antenatal care visit utilization was the commonest significant predictor of perinatal mortality among live births in Ethiopia. Thus, women who had antenatal care visit were 80% [POR = 0.20 (95% CI 0.12, 0.34)] less likely to have perinatal mortality than those women haven’t any antenatal care visit during pregnancy. A random-effect model was used due to significant heterogeneity (I^2^ = 86.7%, *p* value < 0.05 (Fig. 5). The meta-analysis of three studies revealed that maternal tetanus toxoid immunization uptake was another maternal intervention to reduce the perinatal mortality, women who had utilized the tetanus toxoid immunization were 57% [POR = 0.43 (95% CI 0.24, 0.77)] decreased the odds of perinatal mortality than those women who haven’t utilized tetanus toxoid immunization during pregnancy or postpartum period (Fig. 6).

**Figure 7:**
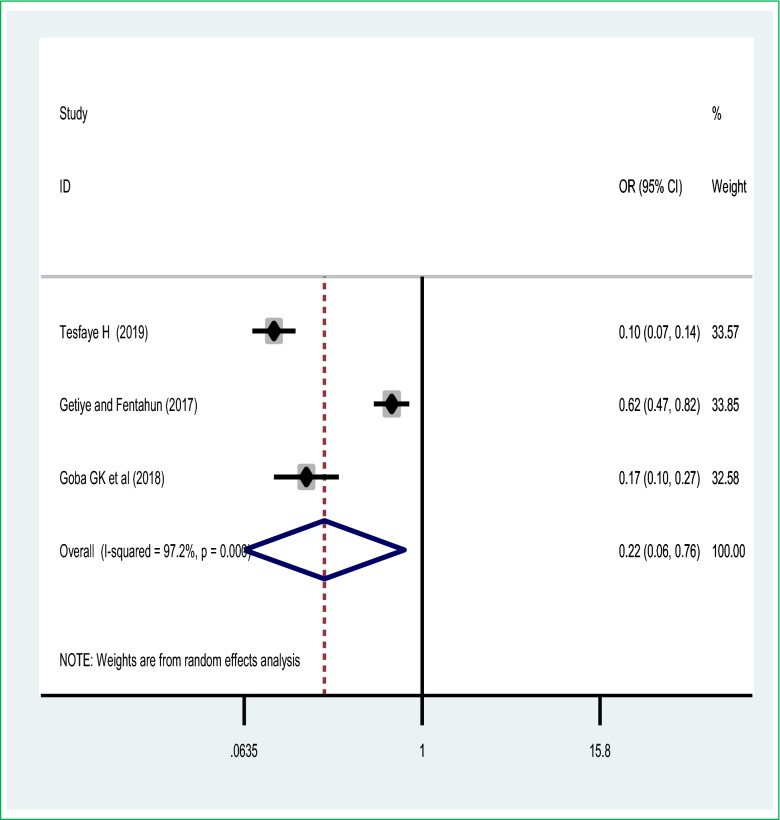
Association of impact of partograph utilization and perinatal mortality.

Also, this systematic review and meta-analysis showed the pooled effect of the most important intrapartum interventions during labour, partograph utilization for monitoring of labour was significantly associated with perinatal mortality; the utilization of partograph for monitoring of labour during the active phase of labour was 78% [POR = 0.22 (95% CI 0.06, 0.76)] less likely to have the chance of perinatal mortality than their counterparts based on the random-effect of a meta-analysis of due to the presence of heterogeneity (I^2^ = 97.2%, with *p* value < 0.0001 (Fig. 7). There was no publication bias based on the Eggers and Beggs test with a *p* value of < 0.05.

## Association of poor maternal obstetric history and perinatal mortality

**Figure 8:**
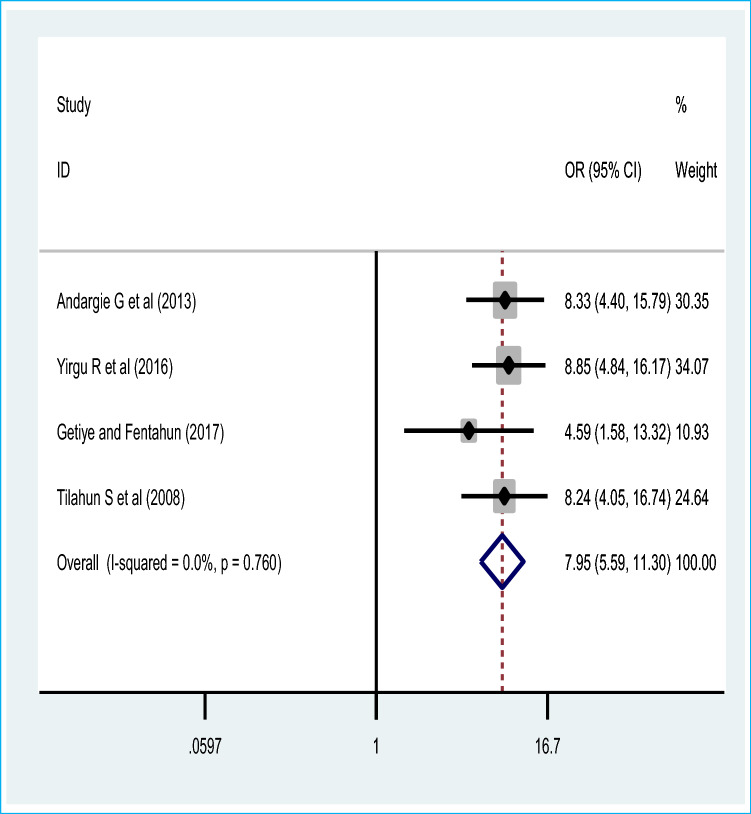
Pooled odds ratio on the association of previous perinatal death and perinatal mortality.

**Figure 9:**
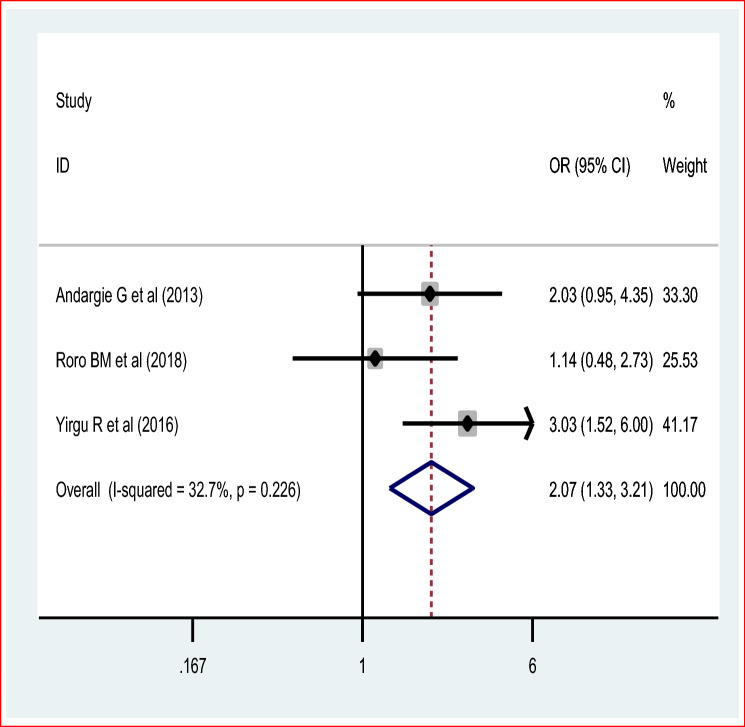
Pooled odds ratio on association of previous abortion and perinatal mortality.

Seven studies were included to assess the effect of poor obstetric history mainly previous perinatal death (based on four studies) and previous abortion history with three studies. Therefore, the pooled effect of the four studies revealed that women who have previous perinatal death were 8 times [OR = 7.95 (95% CI 5.59, 11.30)] more likely to have recent perinatal mortality than those who haven’t any perinatal mortality in previous pregnancy and childbirth. The fixed effect model of meta-analysis was used due to the absence of statistically significant evidence of heterogeneity, I^2^ = 0% and p = 0.76 (Fig. 8). Egger’s test showed a non-significant publication bias. Moreover, the pooled effect of three studies showed that women who have a previous history of abortion were two [OR = 2.07 (95% CI 1.33, 3.21)] times more likely to the perinatal mortality compared with their counterparts. The fixed effect model of the meta-analysis was used due to the lower or absence of evidence of significant heterogeneity (I^2^ = 32.5% and p = 0.23) (Fig. 9). There was no publication bias based on egger’s test.

## Discussions

The systematic review and meta-analysis found that the pooled estimate prevalence of perinatal mortality was 6% (95% CI 5.00, 7.00); which is higher than the study done in SSA, 3.47%^6^. The possible justification for this might be due to the variability in the use and quality of maternal health care services provided to pregnant women and their newborns and the study design difference, in that of the SSA the meta-analysis was done based on the demographic health survey of data of 21 countries, and also the higher burden of cesarean delivery in our setting, and home delivery might be also increased the estimate of perinatal mortality. Hence, a systematic review and meta-analysis supported that rate of perinatal deaths following cesarean sections are disproportionately high in LMICs^56^ and perinatal mortality varies by place of delivery; perinatal mortality is 21% higher for the home compared to facility-based deliveries^57^. Therefore, proper auditing of cesarean delivery to reduce to the optimal recommended level and improving health facility delivery should be emphasised. This meta-analysis also showed that that the pooled prevalence of stillbirth was, 5% (95% CI 3.00, 6.00) which was comparable with the global network study at LMICs with the epidemiology of stillbirth of 3.2%^58^. On the other hand, the current pooled estimate of stillbirth was lower than the findings done in Guatemala, 19.9% and 25.3% in India^59,60^. This variation might be due to the difference in methodology, in the study design and participants included in this systematic review and the countries else.

The findings of this meta-analysis also found that the highest prevalence of perinatal mortality has occurred in Harar and SNNPR and the lowest was at Oromia and Addis Ababa next to the National level. The possible reason for the regional variation in the level of perinatal mortalities in Ethiopia could be explained by the difference in maternal health care service utilization. Hence, a national demographic health survey report in Ethiopia supported that the lowest and highest antenatal care (ANC) service utilization were spatially clustered in Harar (%), SNNPR (39.8%) and Addis Ababa (94%), respectively^61^. Hence, a systematic review supports that ANC visit significantly increases birth in the health facility, which reduces the risk of perinatal mortality^62^. Regarding the lower pooled estimate of perinatal mortality at the national level might be explained due to the inclusion of demographic health survey data, intermittent nature of data collection of demographic health survey data, absence of supply chain information and women’s potential of recall bias, substantially underestimate mortality.

This systematic review and meta-analysis revealed that antenatal care visit is the most significant protective factor for the reduction of perinatal mortality. This finding is in line with other studies done in LMICs^63^, and United States^64^ found that the odds of perinatal mortality increases among women who have no ANC visit. The possible explanation for this might be due to the fact, those women who have ANC visit might be more likely to increase the maternal literacy, early awareness of pregnancy complications and get the continuum care ; a skilled birth attendant at the health facility and Birth Preparedness and Complication Readiness (BPCR) interventions, getting an antenatal screening of complications, intrapartum, postpartum and neonatal care, associated with an increased likelihood of use of care in the event of newborn illness, and initiation of breastfeeding in the first hour of life, encourages to take decisions before the onset of labour and occurrence of obstetric complications and got the service timely^65,66^; reduces of perinatal mortality. Hence, a meta-analysis supports that antenatal screening of antenatal syphilis^67^, gave birth at the health facility^57^ and BPCR interventions^68^ are known factors for the reduction of perinatal mortality. Again, those women who had ANC visit might more likely adhere to prenatal maternal nutrition like iron-folic acid supplementations and other micronutrients, which reduces maternal anaemia that increase the risk of perinatal morbidity, preterm births and low birth weight are the leading cause of perinatal mortality^69,70^. Iron and folic acid and other micronutrients are vital for the development of the fetus, prevents poor birth outcomes. Hence, a meta-analysis revealed that adherence to IFA supplementation and micronutrients is means of reduction of anemia, hemorrhagic newborn disease, congenital anomalies for the fetus, further substantially reduces the risk of perinatal mortality^71,72^. Thus, there remained activities for the government, researchers and organizations in increasing the ANC visit care based on the WHO recommendations within the national context.

Accordingly, this systematic review and meta-analysis found that the immunization of maternal tetanus toxoid was also another predictor that decreases the chance of perinatal mortality. This can be explained because those women who have got the immunization might be less likely to acquire the infection mainly maternal and neonatal tetanus^73^. Maternal and neonatal tetanus causes about half of all perinatal deaths and neonatal mortality today, claiming about 180,000 lives worldwide every year mainly in developing countries. However, it is easily prevented by maternal immunization with tetanus toxoid vaccine during antenatal care visit and the interventions to screen and manage infections during pregnancy and intrapartum period are means of reducing those perinatal mortalities attributable to infection^74^. Despite, the tetanus toxoid vaccination uptake is low, 14.8% to 39%^75,76^ and the dropout rate of the immunization is increasing up to 72.3%^77^ Ethiopia. Thus, future strategies to improve the vaccination rate should be emphasised and future prospective studies have to done to assess the effect of the dose of maternal tetanus toxoid immunization on perinatal outcomes.

This meta-analysis also found that partograph monitoring of labour was another intrapartum intervention that significantly reduces the rate of perinatal mortality. This might be since partograph helps the health care provider in identifying the slow progress of labour and provides an early warning system for early referral and may also help to initiate appropriate interventions within a timely manner. Hence, proper partograph utilization improves labour outcomes^78–81^. Thus, proper partograph monitoring of labour should be improved at the national level to reduce the rate of perinatal mortality and poor labour outcomes. Therefore, a continuum of care has the potential to scale up to improve the maternal, newborn, and child health (MNCH) by ensuring care for mothers and children, indicates that obstetric care needs still to be strengthened, should include the continuum of care from home to the health facility, make care accessible to all, and reduce delays.

Moreover, the results of this systematic review and meta-analysis investigated that women who had a history of previous perinatal mortality significantly increased the odds of perinatal mortality than their counterparts. This was supported by a meta-analysis done at high-income countries^82^. Furthermore, this systematic review and meta-analysis found that a previous history of abortion also significantly increased the chance of having perinatal mortality, which was also supported by previous studies done by Brown et al.^83^ and Saraswat L et al.^84^. The possible reason for the significant increase of perinatal mortality among those women might be more likely to develop maternal stress associated with the previous exposure. Maternal stress increased the risk of preterm birth^85,86^ through increased production of proinflammatory cytokines^87^, cause the production of prostaglandins^88^, weaken fetal membranes and ripen the cervix^89^ increases preterm birth, increased the risk of perinatal death. Thus, improving the care provision, integrating mental health with women with previous bad obstetric history is an area of improvements in future studies.

Despite, there was a very extensive systematic search and inclusion of articles without specifying the period of publications, the results of this review should be interpreted with some limitation. The high heterogeneity in between studies might lead to insufficient statistical power to detect significant association. Thus, a meta-regression analysis revealed that there was no variation due to sample size and publication year. This meta-analysis was also unable to assess the association between the doses of maternal tetanus immunizations with perinatal mortality, an area of future research. Also, the studies included in this review were from only five regions and some studies have a small sample size that might reduce its representative for the country. It was impossible to establish the cause-and-effect relationship.

## Conclusions

A significant number of live births in Ethiopia are suffering from perinatal mortality. Therefore, the antenatal and intrapartum interventions as antenatal care visit, maternal tetanus toxoid vaccination uptake, monitoring of labour with partograph and identifying of the maternal obstetric history should be an area of improvements to reduce perinatal mortality. The Federal Ministry of Health and other concerned bodies should work towards the reduction of perinatal mortality by emphasizing the identified modifiable factors.

## Supplementary Information

Supplementary Information 1.

Supplementary Information 2.

Supplementary Information 3.

Supplementary Legends.

## Data Availability

Data will be available from the corresponding author upon reasonable request.

## Abbreviations

ANC: Antenatal care
BPCR: Birth preparedness and complication readiness
LMICs: Low and middle-income countries
NOS: Newcastle-Ottawa scale
POR: Pooled odds ratio
SSA: Sub Saharan Africa
