# Supplementary material for: Perinatal mortality and its association with antenatal care visit, maternal tetanus toxoid immunization and partograph utilization in Ethiopia: a meta-analysis
Source: Sci Rep. 2021 Oct 4;11:19641. doi: 10.1038/s41598-021-98996-5 (PMC8490438; doi:10.1038/s41598-021-98996-5)
Supplement: Supplementary file 2 — Supplementary Information 2. [file 41598_2021_98996_MOESM2_ESM.docx]

# Perinatal mortality and its association with antenatal care visit, maternal tetanus toxoid immunization and partograph utilization in Ethiopia: a meta-analysis

Melaku Desta *^1^,Tadesse Yirga Akalu ^2^, Yoseph Merkeb Alamneh ^3^, Asmare Talie^1^, Addisu Alehegn *Alemu*^1^, Zenaw Tessema^4^, Dessalegn Yibeltal^4^, Alehegn Aderaw Alamneh^5^, Daniel Bekele Ketema^6^, Wondimeneh Shibabaw Shiferaw ^7^, Temesgen Getaneh ^1^

Supplementary file 2: sensitivity analysis on the pooled prevalence of perinatal mortality
