# Supplementary material for: Perinatal mortality and its association with antenatal care visit, maternal tetanus toxoid immunization and partograph utilization in Ethiopia: a meta-analysis
Source: Sci Rep. 2021 Oct 4;11:19641. doi: 10.1038/s41598-021-98996-5 (PMC8490438; doi:10.1038/s41598-021-98996-5)
Supplement: Supplementary file 3 — Supplementary Information 3. [file 41598_2021_98996_MOESM3_ESM.docx]

# Perinatal mortality and its association with antenatal care visit, maternal tetanus toxoid immunization and partograph utilization in Ethiopia: a meta-analysis

Melaku Desta *^1^,Tadesse Yirga Akalu ^2^, Yoseph Merkeb Alamneh ^3^, Asmare Talie^1^, Addisu Alehegn *Alemu*^1^, Zenaw Tessema^4^, Dessalegn Yibeltal^4^, Alehegn Aderaw Alamneh^5^, Daniel Bekele Ketema^6^, Wondimeneh Shibabaw Shiferaw ^7^, Temesgen Getaneh ^1^

**Supplementary file 3: Quality assessment of included studies**

| **Authors** | **Quality assessment criteria** | | | |
| --- | --- | --- | --- | --- |
|  | **Selection** | **Comparability** | **Outcome** | **Overall quality** |
| Tessema F et al | **** | * | ** | 7 |
| W/Amanuel & Gelebo | **** | * | ** | 7 |
| Aragaw YH | **** | ** | ** | 8 |
| Roro BM et al | **** | * | ** | 7 |
| Tesfaye H | **** | ** | ** | 8 |
| Mihretu A et al | **** | ** | ** | 9 |
| Andargie G et al | **** | ** | ** | 8 |
| Yirgu R et al | **** | ** | ** | 8 |
| Tura AK et al | **** | ** | ** | 8 |
| Debelew GT et al | **** | * | ** | 7 |
| Limaso AA et al | **** | * | ** | 7 |
| Eyowas FA et al | **** | ** | ** | 8 |
| Lolaso T et al | **** | * | ** | 7 |
| Mehari M et al | **** | * | ** | 7 |
| Bayou & Berhan | ***** | ** | ** | 9 |
| Tsegaye and Kassa | **** | * | ** | 7 |
| Desta NB et al | **** | ** | ** | 8 |
| Lakew D et al | **** | * | ** | 7 |
| Adane AA et al | **** | * | ** | 7 |
| Berhe KA et al | *** | * | ** | 6 |
| Assefa N et al | *** | * | ** | 6 |
| Adhena T et al | **** | ** | ** | 8 |
| Abdo RA et al | **** | ** | ** | 8 |
| Cherie & Mebratu | *** | * | ** | 6 |
| Zerfu TA et | *** | * | ** | 6 |

The Newcastle Ottawa Scale (NOS) was used **t**o assess the quality of included

** Two points, *** Three points; and **** four point
