# Supplementary material for: Perinatal mortality and its association with antenatal care visit, maternal tetanus toxoid immunization and partograph utilization in Ethiopia: a meta-analysis
Source: Sci Rep. 2021 Oct 4;11:19641. doi: 10.1038/s41598-021-98996-5 (PMC8490438; doi:10.1038/s41598-021-98996-5)
Supplement: Supplementary file 4 — Supplementary Legends. [file 41598_2021_98996_MOESM4_ESM.docx]

**Supplementary files**

Supplementary file 1: PRISMA checklist

Supplementary file 2: sensitivity analysis on the pooled prevalence of perinatal mortality

Supplementary file 3: Quality assessment of included studies
